# AKT2 drives cancer progression and is negatively modulated by miR-124 in human lung adenocarcinoma

**DOI:** 10.1186/s12931-020-01491-0

**Published:** 2020-09-01

**Authors:** Ting Liu, Jianjie Zhu, Wenwen Du, Weiwei Ning, Yang Zhang, Yuanyuan Zeng, Zeyi Liu, Jian-An Huang

**Affiliations:** 1 Department of Respiratory Medicine, the First Affiliated Hospital of Soochow University, Suzhou, 215006 China; 2 Suzhou Key Laboratory for Respiratory Diseases, Suzhou, 215006 China; 3 Institutes of Biology and Medical Sciences, Soochow University, Suzhou, 215123 China; 4 Institute of Respiratory Diseases, Soochow University, Suzhou, 215006 China

**Keywords:** Lung adenocarcinoma, AKT2, Proliferation, Migration, Invasion, miR-124

## Abstract

**Background:**

AKT2 is highly expressed in many human cancers, including non-small cell lung cancer (NSCLC). Accumulating evidence has also revealed that AKT2 can promote NSCLC cell proliferation and metastasis. However, the involved mechanism remains unclear. Herein, our study mainly explored the function of AKT2 during cancer progression and uncovered a new post-transcriptional mechanism of AKT2 expression in lung adenocarcinoma (LUAD).

**Methods:**

Quantitative real-time (qRT-PCR), western blot and immunohistochemistry (IHC) assays were performed to detect the expression of AKT2 and other proteins. Cell counting kit-8 (CCK-8), colony formation and EdU assays were performed to assess cell proliferation. Flow cytometry analysis was used to detect changes in the cell cycle and apoptosis. Transwell assays were used to evaluate cell migration and invasion. Additionally, a luciferase reporter assay and western blotting were employed to assess miR-124 targeting of AKT2. Xenograft mouse model was used to observe the role of miR-124/AKT2 axis on the occurrence and development of LUAD.

**Results:**

We showed that AKT2 was highly expressed in NSCLC tissues and closely related to the poor prognosis of LUAD patients. Moreover, AKT2 affected LUAD cell proliferation, migration and invasion by regulating the cell cycle and promoting the occurrence of epithelial-mesenchymal transition (EMT) and the expression of matrix metalloproteinases (MMPs). In addition, we demonstrated that miR-124 overexpression downregulated AKT2 expression by binding to the 3′-untranslated region (3′- UTR) of AKT2 and thus inhibited the occurrence and development of LUAD in vivo and in vitro*.*

**Conclusions:**

Our results suggest that miR-124 overexpression can negatively regulate AKT2 and thus inhibit the progression of LUAD. Therefore, the miR-124/AKT2 axis may serve as a potential target for novel therapies for LUAD.

## Introduction

Lung cancer has the highest mortality rate among cancer-related diseases based on the latest worldwide report [1]. Non-small cell lung cancer (NSCLC) accounts for approximately 85% of cases among 1.8 million newly diagnosed patients [1, 2]. Lung adenocarcinoma (LUAD) and squamous cell carcinoma (LUSC) are two main subtypes. Despite advances in the diagnosis and treatment of NSCLC, the five-year survival rate is still poor [3]. Therefore, a deeper understanding of the pathogenesis of lung cancer is urgently needed.

AKT (protein kinase B, PKB) family members are well known for their roles in regulating tumorigenesis and development [4]. AKT1, AKT2 and AKT3 belong to the AKT family. All the AKT family members contain similar protein structures, such as the N-terminal, PH domain, catalytic domain and C-terminal regulatory domain, although the members are encoded by different genes [5]. Tissue distributions are varied among these family members. AKT1 and AKT2 are widely expressed in human tissues, while AKT3 is mainly distributed in brain tissue [4]. Among these three, AKT2 is much more closely associated with cancer cell metabolism, proliferation, cell survival, metastasis, angiogenesis and drug resistance [6]. In breast cancer, inhibition of AKT2 can not only effectively prevent the transformation of mesenchymal non-cancer stem cells (non-CSC) into epithelial cells but also reduce the invasive and colony formation abilities of non-CSC and CSC [7]. In colon cancer, the expression of AKT2 can affect the DNA repair ability and radiosensitivity [8]. In NSCLC, AKT2 can affect tumor cell survival and chemotherapy sensitivity [9]. Thus, AKT2 is considered a promising target for cancer-targeted therapy.

MicroRNAs are single strand small noncoding RNAs that partially or completely bind to the 3′-UTR region of target mRNAs to modulate gene expression, resulting in the regulation of proliferation, differentiation, apoptosis and metastasis in cancer cell progression [10, 11]. MiR-124 expression has been reported to be widely downregulated in many cancers, such as breast cancer, colon cancer, glioma, lymphoma, NSCLC and so on [12–16]. Accumulating reports have demonstrated the roles of miR-124 in the occurrence and development of a variety of tumors. In neuroblastoma, miR-124 induces neuroblastoma differentiation by downregulating the expression of the transcription factor ELF4 [17]. In bladder cancer, miR-124 negatively modulates EDNRB to suppress proliferation and induce apoptosis in tumor cells [18]. Although miR-124 expression was suggested to be downregulated in NSCLC, the molecular mechanism underlying the involvement of the miR-124/AKT2 axis in mediating cell proliferation and metastasis is not fully understood, especially in LUAD subtypes [16, 19, 20].

In our current study, we proved that AKT2 expression was upregulated in NSCLC tissues. Knockdown of AKT2 expression could attenuate cell proliferation via cell cycle arrest. Moreover, migration was also inhibited by reversing the EMT process and regulating the MMP family. In addition, we confirmed that miR-124 could directly target the 3′-UTR of AKT2 and thus inhibit AKT2 expression in vivo and in vitro. Taken together, our data identify a novel mechanism by which the miR-124/AKT2 axis mediates carcinogenesis and provide new potential therapeutics for LUAD.

## Materials and methods

### Patients and tissue samples

Tissue samples from 45 NSCLC patients and matched noncancerous tissue samples collected between 2012 and 2016 were obtained from the First Affiliated Hospital of Soochow University. All cases were confirmed by experienced clinicians and pathologists, and no patient received any relevant treatment before sampling. All collected samples were stored at − 80 °C. Each patient involved in the research signed a written informed consent form, and this study was approved by the Ethics Committee and Institutional Review Board of the First Affiliated Hospital of Soochow University. All the methods used in the study are based on the approved guidelines.

### Immunohistochemisty (IHC) staining

Tissue samples were fixed with 4% paraformaldehyde and embedded in paraffin. After baking, deparaffinization and rehydration, slides were immersed in 3% H_2_O_2_ for 15 min, heated at 95 °C for 25 min and cooled at room temperature. These sections were then incubated with an anti-AKT2 polyclonal antibody (diluted 1:200, Proteintech, 17,609–1-AP) and an anti-KI67 antibody (diluted 1:400, Cell Signaling Technology, 9449) at 4 °C overnight and visualized using diaminobenzidine as the chromogenic substrate (DAB kit, ZSGB-Biotechnology Co., Ltd., Beijing, China). Finally, the sections were counterstained with hematoxylin and examined by optical microscopy. The staining results for AKT2 were evaluated based on the staining intensity and the percentage of positively stained cells.

### Cell culture

Cells were provided by the Cell Bank of the Chinese Academy of Sciences (Shanghai, China). RPMI 1640 medium supplemented with 10% fetal bovine serum (Gibco, Carlsbad, CA, USA), penicillin (100 U/ml) and streptomycin (100 ng/ml) was used for cell culture. All cell lines were maintained in 37 °C humidified incubators with 5% CO_2_.

### Western blot analysis and antibodies

Western blot analysis was performed as we described previously [21]. In this study, the antibodies used were anti-AKT2 (17609–1-AP, Proteintech, China); anti-pAKT (Ser473) (D9E), anti-AKT, anti-pErk (Thr202/Tyr202) (D13.14.4E), anti-Erk (137F5), anti-Slug (C19G7), anti-MMP9 (603H), anti-MMP7 (D4H5), and anti-MMP2 (D8N9Y) (Cell Signaling Technology, Danvers, MA, USA); anti-N-cadherin and anti-vimentin (RV202) (BD Biosciences, USA); and anti-β-actin and anti-mouse or anti-rabbit secondary antibodies (Cell Signaling Technology). Each experiment was performed in triplicate.

### RNA isolation and quantitative real-time PCR analysis

The detailed processes were performed as we previously described [22]. The primer sequences used in this study are listed in Supplementary Additional file 1 Table S1 (Sangon Biotech, Shanghai, China). MiR-124 and U6 primers were purchased from RiboBio Co. (Guangzhou, China). The CT values of *AKT2* mRNA and miR-124 were normalized to those of *ACTB* mRNA and U6, respectively. The ^△△^Ct method was applied to calculate the relative quantities of these mRNAs. Each experiment was performed in triplicate.

### MicroRNA (miRNA) and small interfering RNA (siRNA) transfection

A549 and H1299 cells were seeded in the 6-well plates. When the cell intensity reached 60%, we replaced the medium with 1.5 ml fresh serum-free RPMI-1640 medium for 2 h and then 250 μl serum-free medium containing 5 μl Lipofectamine 2000 transfection reagents or 5 μl siRNA /microRNA (50 nmol per well) were mixed for 15 min at room temperature. Later, the above mixture was added into 6-well plates. Six hours later, the cell supernatant was changed with 2 ml medium with 10%FBS. After 48–72 h transfection, the cells were collected for further experiments. MiR-124 mimic and negative control were purchased from GenePharma (Shanghai, China). The siRNAs specific for AKT2 were provided by Shanghai GenePharma Company. The target sequences of the siRNAs were as follows: siRNA-AKT2–1: 5′-GCUCCUUCUAUUGGGUACAATT-3′, siRNA-AKT2–2: 5′-GCGGAAGGAAGUCAUCAUUTT-3′, and siRNA-AKT2–3: 5′-GGUUCUUCCUCAGCAUCAATT-3′.

### CCK-8, colony formation and EdU assays

Cell proliferation was evaluated using Cell Counting Kit-8 (Beyotime Institute of Biotechnology). Briefly, cells were cultured in 96-well plates and seeded at 3000 cells per well. At 24 h, 48 h and 72 h, 10 μl CCK-8 dye was added to each well and incubated at 37 °C for 3–4 h. Then, the absorbance was measured at 450 nm and 630 nm using a spectrophotometer (Thermo Fisher Scientific). For the colony formation assay, cells were cultured for 7–10 days until foci formed. The cells were then fixed with methanol and stained with crystal violet. For the EdU assay, we performed the experiment according to the kit instructions. A cell-light EdU Apolo567 in vitro kit was purchased from RiboBio Co. The experiments were performed in triplicate.

### Cell migration and invasion assays

For the migration assay, 3 × 10^4^ transfected cells suspended in medium containing 1% FBS were added to the upper chamber, and 800 μl normal medium containing 10% FBS was added to the lower chamber. For the invasion assay, the upper chamber was coated with a Matrigel matrix at a 1:6 dilution (BD Science, Sparks, MD, USA). Then, 1% FBS medium containing 5 × 10^4^ tumor cells was added to the upper chamber, and 800 μl 10% FBS medium was added to the lower chamber. After culturing for 24 h, the cells were fixed with methanol, stained with crystal violet, and imaged under a microscope. The transwell chambers used in our study were purchased from BD Biosciences. Each experiment was performed independently in triplicate.

### Flow cytometry analysis

For cell cycle assay, transfected tumor cells were washed with PBS, suspended in 70% ethanol and then fixed at 4 °C overnight. Then, the cells were stained with a mixed propidium iodide solution and incubated at 37 °C for 30 min. For apoptosis assay, transfected tumor cells were stained with an Annexin V/PI kit (Beyotime, Shanghai, China). Finally, the cells in both experiments were evaluated using a fluorescence-activated cell sorting (FACS) Caliber system (Beckman Coulter, Brea, CA, USA). Each experiment was performed independently in triplicate.

### Dual-luciferase reporter assay

143-bp sequence of the 3′-UTR of AKT2 containing the putative miR-124 binding site or mutated target site were synthesized and cloned into the pGL3 basic vector (Promega, Madison, WI, USA) which were named WT (wild-type AKT2 3′-UTR fragment) and Mut (mutant AKT2 3′-UTR fragment). A549 and H1299 cells were seeded in 24-well plates and co-transfected with WT or Mut plasmids along with either miR-NC or miR-124 mimic using Lipofectamine 2000 (Invitrogen, Carlsbad, CA, USA). At the same time, pRL-TK Renilla Luciferase Reporter Vector (Promega) was transferred into each well. After incubated for 48 h, the cell lysates were harvested. The luciferase activity was assessed by the Dual-Luciferase Reporter Assay Kit (Promega) and then standardized with renilla luciferase activity.. Each experiment was performed independently in triplicate.

### Tumorigenesis in nude mice

Female BALB/c nude mice were obtained and bred in the Experimental Animal Center of Soochow University. In total, 2 × 10^6^ A549 cells were injected subcutaneously into the mice. Fifteen days after inoculation, a miR-124 agomir (RiboBio Co.) and miR-NC agomir were used for intratumoral treatment at a dose of 2 nmol per tumor. Intratumoral treatment was performed three times a week for a total of seven times. Xenograft tumor volume and body weight of mice were measured every 2–4 days for a total of 36 days. We used the volume measurements to evaluate the growth of tumors, and tumor volume was calculated by measuring the length and width of a xenograft tumor and using the following formula: (Volume = length × width^2^)/2.

### Statistical analysis

Student’s t-test was used for statistical analysis, and *P* < 0.05 was considered significant. All statistical analyses were performed using GraphPad Prism 5.0 (GraphPad, San Diego, CA, USA) and SPSS 7.0 software (SPSS, Chicago, IL, USA). All results are presented as the mean ± SD (standard deviation). Kaplan-Meier methods were used for survival analysis, and survival curves were compared by the log-rank test.

## Results

### AKT2 is overexpressed in human NSCLC tissues

**Fig. 1:**
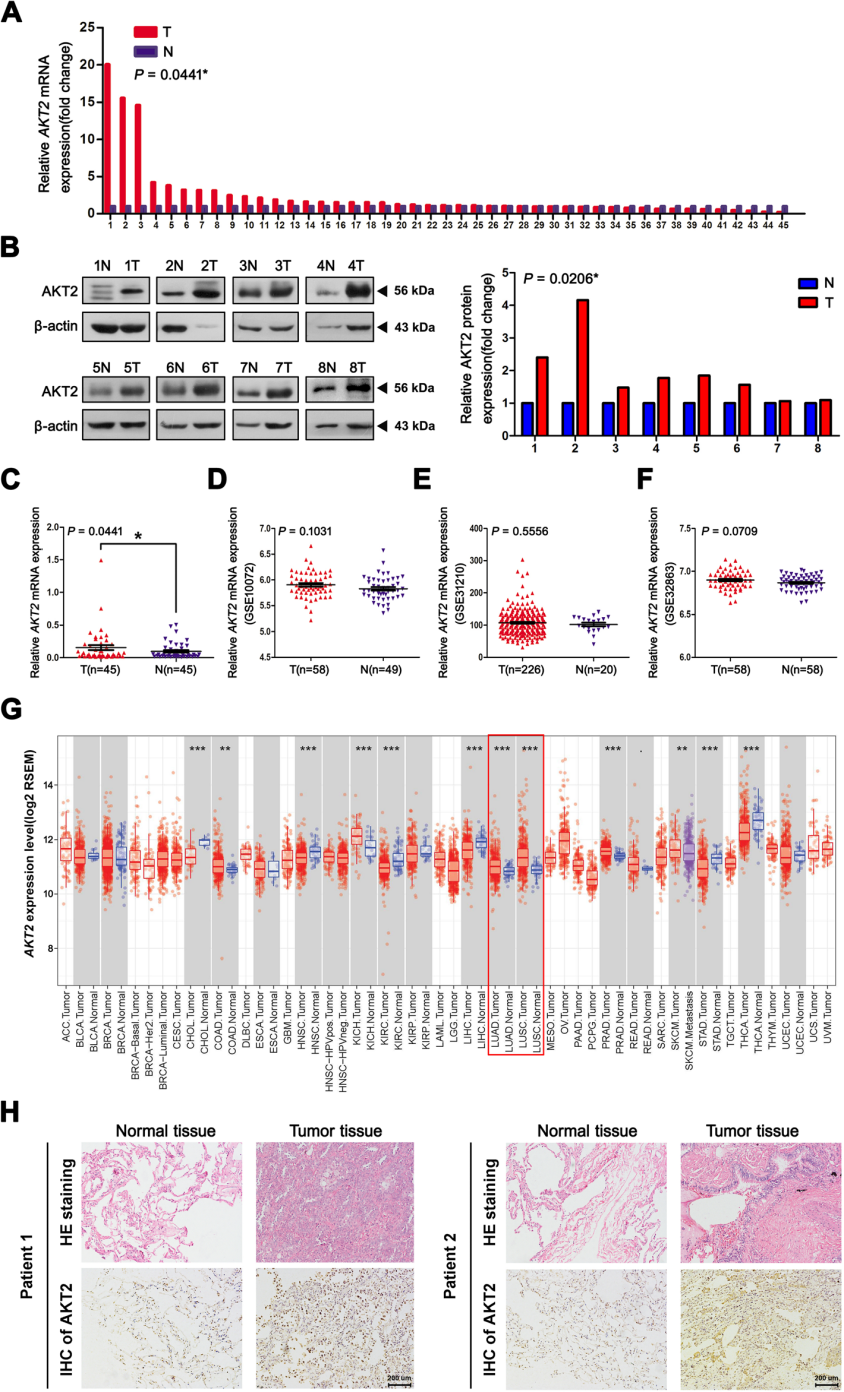
AKT2 is overexpressed in human NSCLC tissues. (**a** and **c**) Relative quantification of AKT2 expression was analyzed by qRT-PCR assay in 45 NSCLC tissues (T) and adjacent noncancerous tissues (N). *ACTB* mRNA levels were used as internal control for normalization of *AKT2* mRNA expression. **b** Western blot analysis was performed to detect the AKT2 protein expression in 8 selected NSCLC tissues and adjacent tissues (left) and the quantification of relative AKT2 protein levels were shown in histogram (right). β-actin was used as internal control. **d**-**f** The public datasets from GEO (GSE 10072, GSE 31210 and GSE 32863) were used to verify *AKT2* mRNA levels in NSCLC. **g** The public data from TIMER database was used to detect *AKT2* mRNA expression in both LUAD tissues and LUSC tissues. **h** Tissue and normal samples from randomly selected patients with NSCLC were either stained with Hematoxylin and eosin (HE) (upper, *n* = 2 per group) or immunohistochemically (IHC) stained with an AKT2 antibody (bottom, *n* = 2 per group). Scale bar, 200 um. T: NSCLC tumor tissues N: Adjacent normal tissues. *P < 0.05; **P < 0.01; ***P < 0.001

First, qRT-PCR analysis was carried out to compare AKT2 mRNA expression between 45 paired NSCLC patient tissues and adjacent normal tissues. Among them, 28 NSCLC tissues exhibited higher AKT2 mRNA expression levels (28/45, 62.22%) than the corresponding paracancerous tissues (Fig. 1a and c; *P* = 0.0441). Western blot analysis also showed higher AKT2 protein levels in 8 paired NSCLC tissues (Fig. 1b, *P* = 0.0206). However, we did not find any significant associations between AKT2 mRNA expression and clinical characteristics including age, histology, lymph node metastasis status, distant metastasis status and TNM stage, but there was a difference in expression between the sexes, which needs to be further explored in large populations (Additional file 2 Table S2). In addition, to further explore the expression of AKT2 in NSCLC tissues, data from the GEO database were analyzed. As shown in Fig. 1d-f, the data extracted from GSE 10072, GSE 31210 and GSE 32863 consistently indicated that there was no significantly statistical difference of AKT2 expression in NSCLC tissues when compared to adjacent tissues, but there was an upward trend of *AKT2* mRNA levels in NSCLC tissues compared with normal tissues. Data from The Tumor Immune Estimation Resource (TIMER) database (https://cistrome.shinyapps.io/timer/) showed that AKT2 was overexpressed in both LUAD and LUSC tissues (Fig. 1g). In addition, an immunohistochemistry assay similarly showed higher AKT2 protein levels in lung cancer tissues than in normal tissues (Fig. 1h). In summary, these results demonstrated that AKT2 expression was upregulated in NSCLC tissues, suggesting that AKT2 acts as an oncogene in NSCLC.

### Increased AKT2 expression is associated with poor prognosis in lung adenocarcinoma patients

**Fig. 2:**
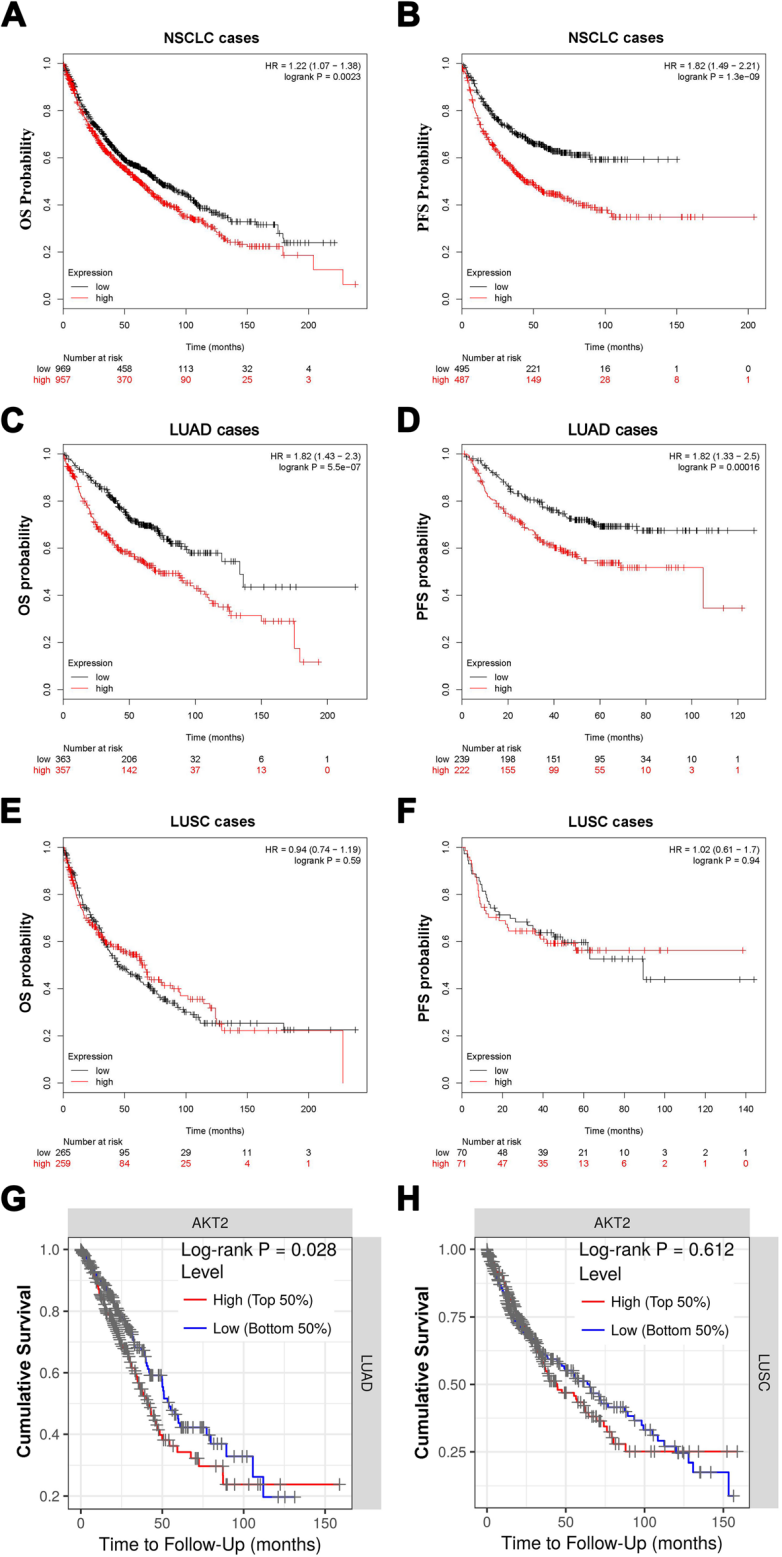
Up-regulated AKT2 expression is associated with poor prognosis in LUAD patients. **a** and **b** Kaplan-Meier plots from Kaplan-Meier plotter shows the correlation between AKT2 expression and overall survival (**a**) or progression-free survival in patients with NSCLC (**b**). **c**-**f** Kaplan-Meier plotter data set shows the correlation between AKT2 expression and the overall survival or progression-free survival in LUAD (**c**, **d**) and LUSC (**e**, **f**) patients. **g** and **h** Public data from TIMER database shows the correlation between AKT2 expression and overall survival in LUAD (**g**) and LUSC (**h**) patients. OS, overall survival; PFS, progression-free survival. *P < 0.05; **P < 0.01; ***P < 0.001

To further explore the prognostic significance of AKT2 in NSCLC, the public database Kaplan-Meier plotter (www.kmplot.com) was used. Data from the database showed that higher expression of AKT2 was associated with poorer overall survival (OS) (Fig. 2a, HR = 1.22, 95% CI: 1.07–1.38, *P* = 0.0023) and progression-free survival (PFS) (Fig. 2b, HR = 1.82, 95% CI: 1.49–2.21, *P* = 1.3e-09) in NSCLC patients. Then, we divided the data into LUAD and LUSC subtypes to further explore the correlation between AKT2 expression and the survival rates in these two subsets of patients. Interestingly, we found that AKT2 overexpression was closely related to poorer OS and PFS in LUAD (Fig. 2c and d; both *P* < 0.05) but not in LUSC (Fig. 2e and f; both *P* > 0.05). Moreover, data from the TIMER database showed results similar to those from the Kaplan-Meier plotter database. When the AKT2 expression level was divided into the top 50% and lower 50%, we found that higher AKT2 expression was significantly correlated with poorer OS in LUAD patients but not in LUSC patients (Fig. 2g and h). Taken together, these results suggested that high AKT2 expression could be regarded as an predictive indicator for poor prognosis in LUAD patients.

### Knockdown of AKT2 expression suppresses tumor cell viability and arrests the cell cycle in LUAD

**Fig. 3:**
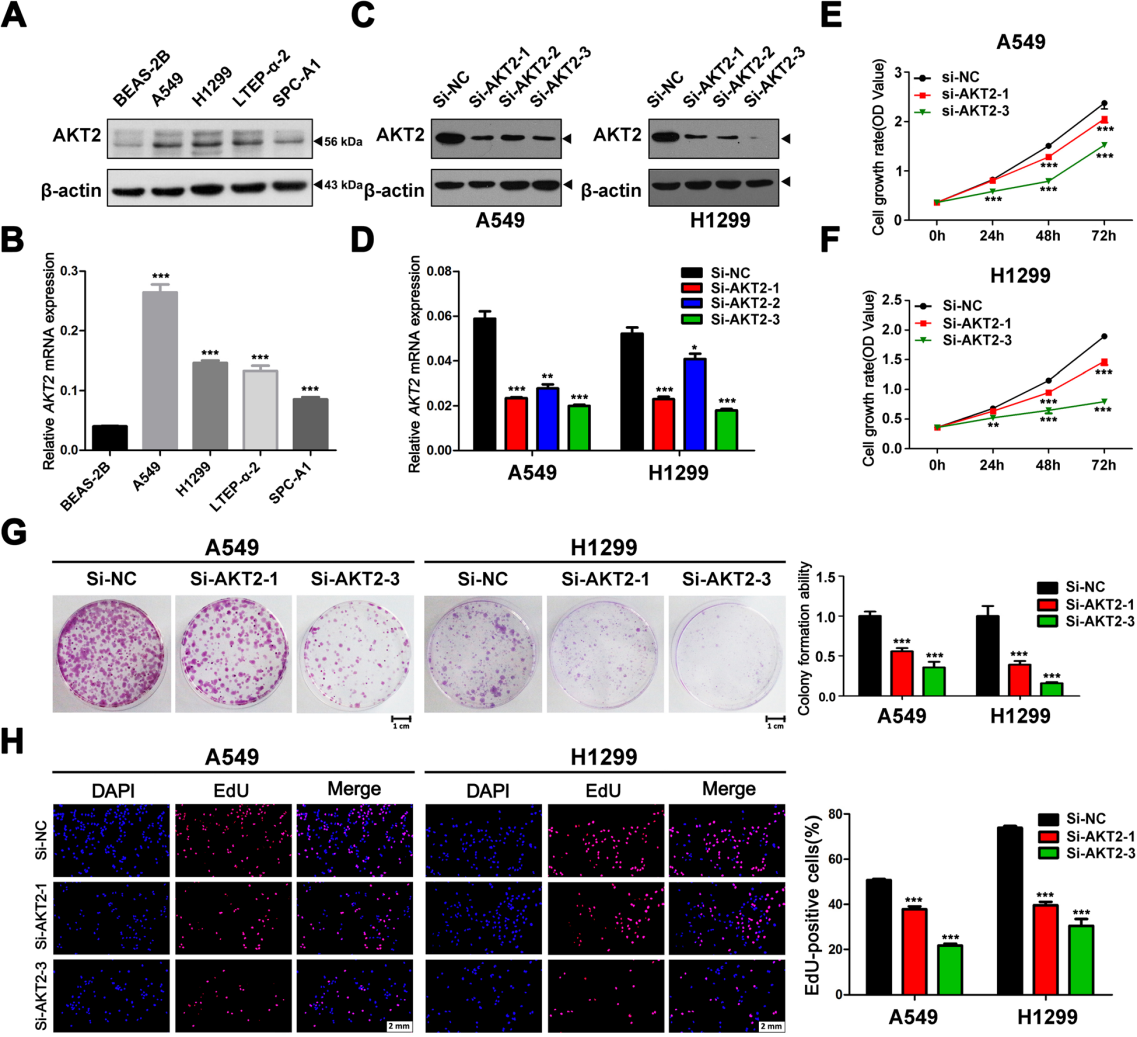
Knockdown of AKT2 significantly prevents cell proliferation in LUAD cell lines. **a** and **b** AKT2 levels were detected by western blot (**a**) and qRT-PCR (**b**) in multiple NSCLC cell lines. **c** and **d** A549 and H1299 cells were transfected with control siRNA or siRNA against AKT2, the effective knock down of AKT2 expression was validated by western blot (**c**) and qRT-PCR (**d**). **e** and **f** CCK-8 assay was performed to detect the cell growth in AKT2 silenced A549 (**e**) and H1299 (**f**) cells. **g** Colony formation abilities were detected in AKT2 silenced A549 and H1299 cells. Representative images of colonies for cell proliferation were shown (left). The colony numbers in AKT2 silenced cells were normalized to negative control (right). **h** An EdU staining assay was performed to determine the proliferation ability of AKT2 silenced A549 and H1299 cells (left). EdU-positive ratio were shown (right). Scale bar, 2 mm. Each experiment was performed in triplicate independently. Student’s t-test was used for statistical analysis and data are presented as the mean ± SD.*P < 0.05; **P < 0.01; ***P < 0.001

**Fig. 4:**
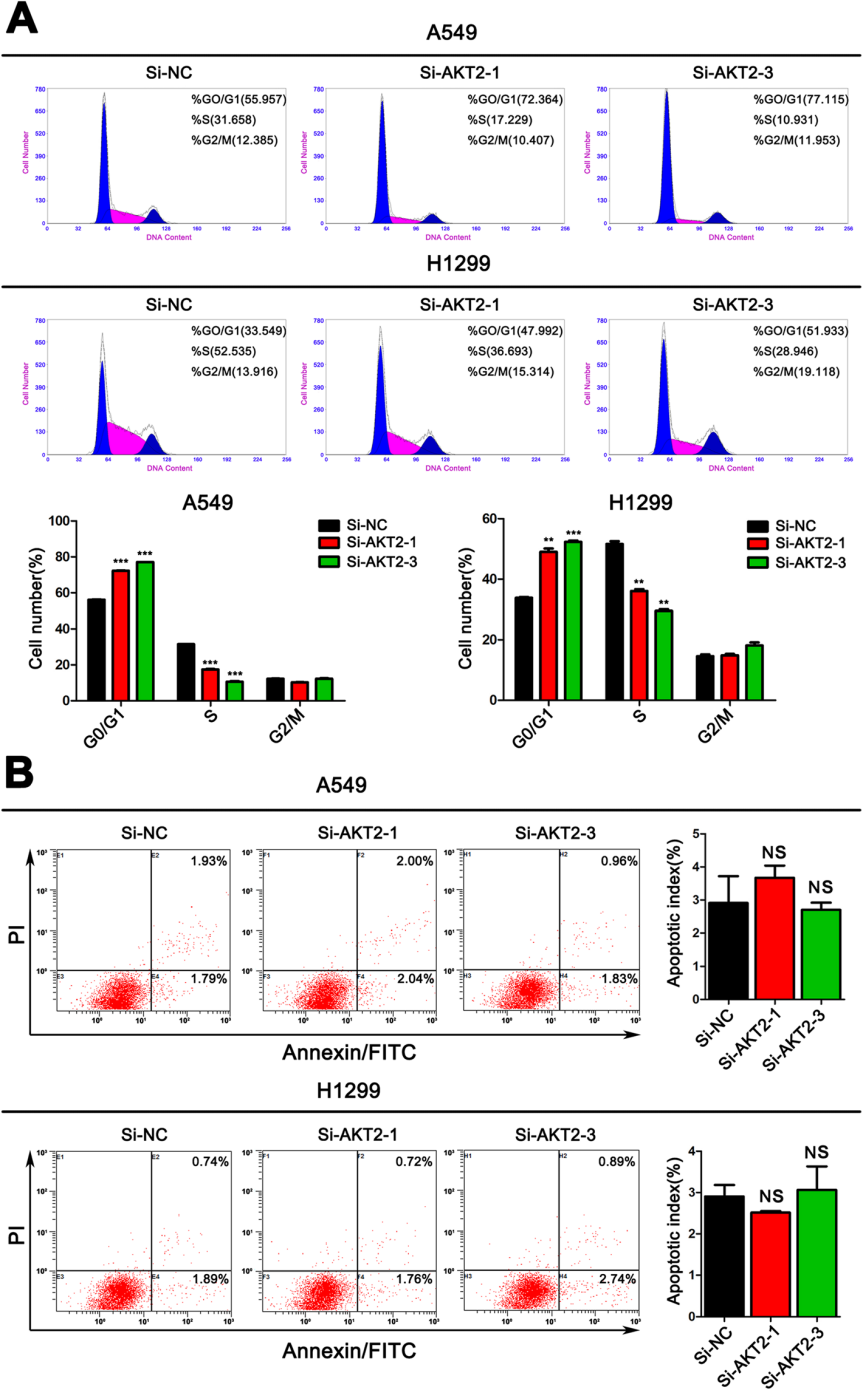
Silencing AKT2 attenuates the cell cycle progression of LUAD cells without affecting apoptosis. **a** Flow cytometry cell cycle analysis was performed in A549 and H1299 cells transfected with Si-NC, Si-AKT2–1 and Si-AKT2–3. The distribution (%) of G0/G1, S and G2/M phases are shown in the histograms (bottom). **b** Flow cytometry apoptosis assay was performed in A549 and H1299 cells transfected with Si-NC, Si-AKT2–1 and Si-AKT2–3. 48 h post-transfection, cells were harvested and stained with Annexin V/FITC and propidium iodide (PI). The percentage of apoptotic cells are shown in the right panel. The date values represent mean ± SD of three measurements independently. NS: no significance. *P < 0.05; **P < 0.01; ***P < 0.001

From the previous clinical results and analysis, we speculated that AKT2 functions as an oncogene in LUAD. To verify this hypothesis, we first detected the AKT2 expression levels in LUAD cell lines. QRT-PCR and western blot assays showed higher AKT2 expression levels in LUAD cell lines than in lung epithelial cells (Fig. 3a and b). Then, we selected A549 and H1299 cells, which expressed relatively high AKT2 levels, for further experiments. Consequently, we silenced the expression of AKT2 with siRNAs) (Fig. 3c and d). CCK-8 and colony formation assays were performed with AKT2-silenced A549 and H1299 cells. The results showed that knockdown of AKT2 expression significantly inhibited tumor cell proliferation (Fig. 3e-g). EdU staining experiments further confirmed that cell proliferation was attenuated after AKT2 knockdown (Fig. 3h). In addition, a flow cytometry assay was performed, and the results demonstrated that the percentage of cells in the G0/G1 phase was increased, and the percentage of cells in the S phase was decreased in AKT2-silenced A549 and H1299 cells (Fig. 4a). However, we did not observe any effect on the apoptosis of tumor cells after inhibiting the expression of AKT2, indicating that AKT2 may control cell proliferation via cell cycle regulation (Fig. 4b).

### Knockdown of AKT2 expression inhibits tumor cell migration and invasion in LUAD

**Fig. 5:**
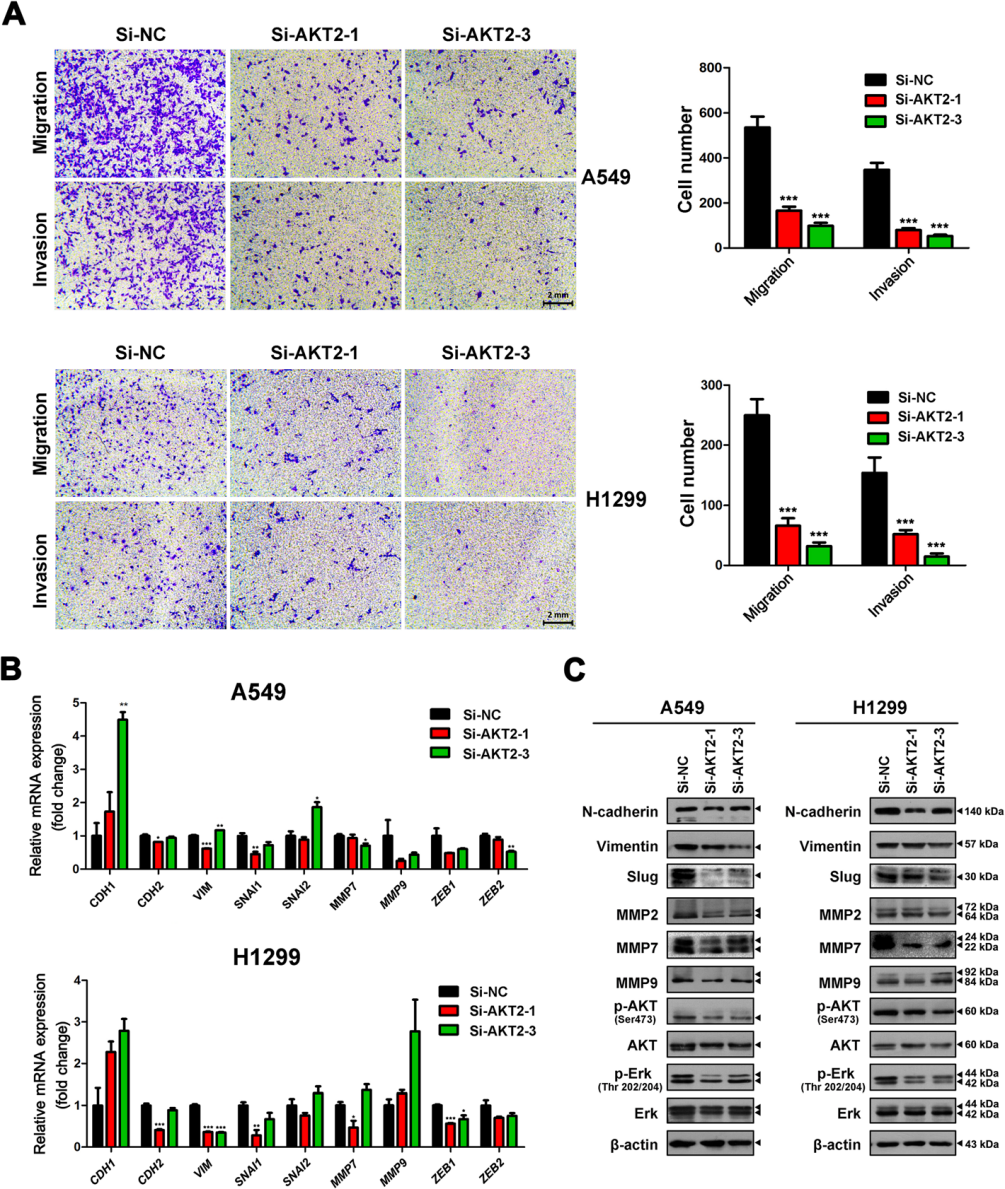
Depletion of AKT2 inhibits cell migration and invasion in LUAD cells. **a** AKT2-silenced A549 and H1299 cells were allowed to migrate through an 8-μM pore in Transwells. The migratory cells were stained and counted in at least three microscopic fields. Cells were then treated as above and allowed to invade through the matrigel-coated membrane in the transwell inserts. Invaded cells were stained and counted under a microscope. Representative images (left) and the migratory cell numbers (right) were shown. Scale bar, 2 mm. **b** The mRNA expression of *CDH1, CDH2, VIM, SNAI1, SNAI2, MMP7, MMP9, ZEB1* and *ZEB2* were detected by qRT-PCR in AKT2 silenced A549 (upper) and H1299 (bottom) cells. **c** The expression of N-cadherin, Vimentin, Slug, MMP2, MMP7, MMP9, AKT, p-AKT, Erk and p-Erk protein levels were examined by western blot in AKT2 silenced A549 (left) and H1299 (right) cells. Each experiment was performed in triplicate independently and values are expressed as mean ± SD. *P < 0.05 **P < 0.01 ***P < 0.001

Transwell assays were used to evaluate the effects of AKT2 on LUAD cell migration and invasion. After transiently silencing AKT2 gene expression, the migratory and invasive abilities of cells were inhibited by more than 50% (Fig. 5a). Next, we used qRT-PCR (Fig. 5b) and western blotting (Fig. 5c) to explore the potential mechanism underlying AKT2-mediated metastasis and proliferation. After transfection of si-AKT2 in A549 and H1299 cells, the expression of not only EMT-related markers (N-cadherin, Vimentin and Slug) but also some matrix metalloproteinases (MMP2, MMP7, and MMP9) was significantly decreased. Additionally, phosphorylated AKT and Erk levels were dramatically decreased in AKT2-silenced A549 and H1299 cells, while the corresponding total protein levels remained unchanged. These results demonstrated that knockdown of AKT2 expression inhibited tumor cell migration and invasion in LUAD.

### AKT2 is a direct target of miR-124 and modulated by miR-124

**Fig. 6:**
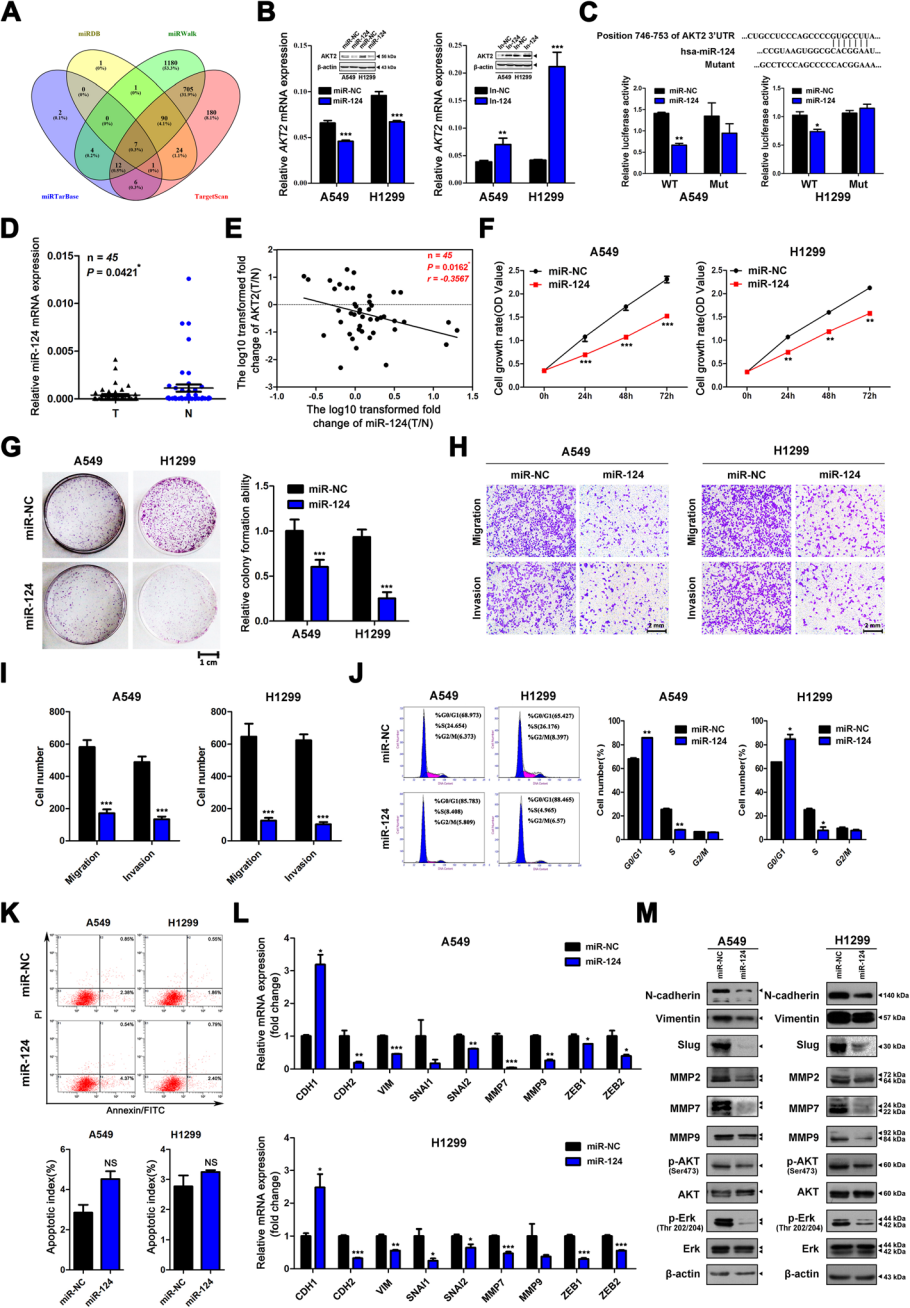
MiR-124 directly binds to AKT2 3’UTR region and thus inhibits LUAD cell proliferation, migration and invasion. **a** Authoritative bioinformatic databases (miRDB, miRWalk, miRTarBase and TargetScan) were used to predict the putative miRNAs targeting AKT2. **b** After transfection with miR-124 mimics and miR-NC (left) or miR-124 inhibitor or inhibitor-NC (right) in A549 and H1299 cell lines, the mRNA and protein levels of AKT2 were analyzed by western blot and qRT-PCR assays. **c** Computational algorithms predict that AKT2 3’UTR region harbors a putative miR-124 binding site (upper). Reporter vector containing wild type AKT2 3’UTR fragment was constructed, and different mutation was then introduced into the potential miR-124 targeting site to obtain mutant type 3′UTR. The generated luciferase reporter plasmids were co-transfected with negative control (miR-NC) or miR-124 mimics into A549 and H1299 cells. The relative firefly luciferase activity was determined and normalized to the Renilla luciferase activity (bottom). **d** qRT-PCR analysis of miR-124 expression in 45 paired NSCLC tissues and adjacent normal tissues. **e** Kaplan-meier analysis was used to detect the correlation between miR-124 and AKT2 expression in 45 paired NSCLC tissues and adjacent normal tissues. miR-124 and *AKT2* mRNA levels are normalized against U6 and β-actin, respectively. *X* and *y* axes represent the log_10_ transformed T/N expression ratios of miR-124 and *AKT2* mRNA, respectively. **f** and **g** CCK8 (**f**) and colony formation (**g**) assays were used to determine the proliferation abilities of miR-124 mimics or negative control (Si-NC) transfected A549 and H1299 cells. **h** and **i** miR-124 overexpressed A549 and H1299 cells were allowed to migrate through an 8-μm pore or invade through matrigel-coated membrane in transwells. Migratory and invasive cells were stained and counted in at least three light microscopic fields. Representative images (**h**) and migratory or invasive cell numbers (**i**) were shown. Scale bar, 2 mm. **j** and **k** Flow cytometry cell cycle analysis of A549 and H1299 cell lines after miR-124 overexpression (left). The distribution (%) of G0/G1, S and G2/M phases are shown in the histograms (right). **l** and **m** Indicated EMT-related mRNAs (**l**) and proteins (**m**) were analyzed by qRT-PCR and western blot in miR-124 overexpressed A549 and H1299 cells. Each analysis was performed in triplicate and Values are represented as means ± SD.*P < 0.05 **P < 0.01 ***P < 0.001

MicroRNA/mRNA interactions are a common mechanism for regulating gene expression. To identify the putative miRNAs that account for the upregulation of AKT2 expression in LUAD, we screened four authoritative bioinformatic databases including miRDB (http://mirdb.org/miRDB/), miRWalk (http://mirwalk.umm.uni-heidelberg.de), miRTarBase (http://mirtarbase.mbc.nctu.edu.tw/php/index.php) and TargetScan (www.targetscan.org) (Fig. 6a). MiR-124, which has been proven to be downregulated in LUAD tissues, was selected [23]. After transfection of miR-124 mimic into A549 and H1299 cells, the expression of AKT2 decreased significantly at both the mRNA and protein levels. In contrast, miR-124 inhibitor transfection induced upregulated AKT2 expression (Fig. 6b). To further confirm that AKT2 is a direct target of miR-124, we synthesized plasmids containing the 3′-UTR region (746–753) of AKT2, which was predicted as a potential binding site. A luciferase assay showed that miR-124 significantly inhibited the relative luciferase activity of A549 and H1299 cells transfected with the wild-type sequence, but there was no effect on cells transfected with the mutated sequence (Fig. 6c). Furthermore, we used qRT-PCR to validate miR-124 expression in 45 paired NSCLC tissues and normal lung tissues. The data showed that miR-124 expression was lower in lung cancer tissues than in adjacent normal tissues (Fig. 6d, *P* = 0.0421). However, depending on the patient data we used, miR-124 expression showed no significant associations with clinical parameters (Additional file 2 Table S2). In addition, the correlation analysis showed that the expression of miR-124 was negatively correlated with that of AKT2 in 45 NSCLC tissues (Fig. 6e, *P* = 0.0162, *r* = − 0.3567).

Then, we investigated the effect of miR-124 on the biological behavior of LUAD cells. CCK-8 and colony formation assays indicated that miR-124 overexpression could significantly inhibit cell proliferation in LUAD (Fig. 6f and g). Flow cytometry analysis showed that overexpression of miR-124 arrested the cell cycle in the G0/G1 phase (Fig. 6j) but had no effect on apoptosis (Fig. 6k). In addition, transwell assays showed that migratory and invasive abilities were inhibited in A549 and H1299 cells transfected with miR-124 (Fig. 6h and i). Finally, we also found that the expression levels of EMT markers, MMPs, p-AKT and p-Erk were significantly decreased at the mRNA and protein levels after miR-124 transfection (Fig. 6l and m).

### MiR-124 overexpression inhibits tumor growth by suppressing the expression of AKT2 in vivo

**Fig. 7:**
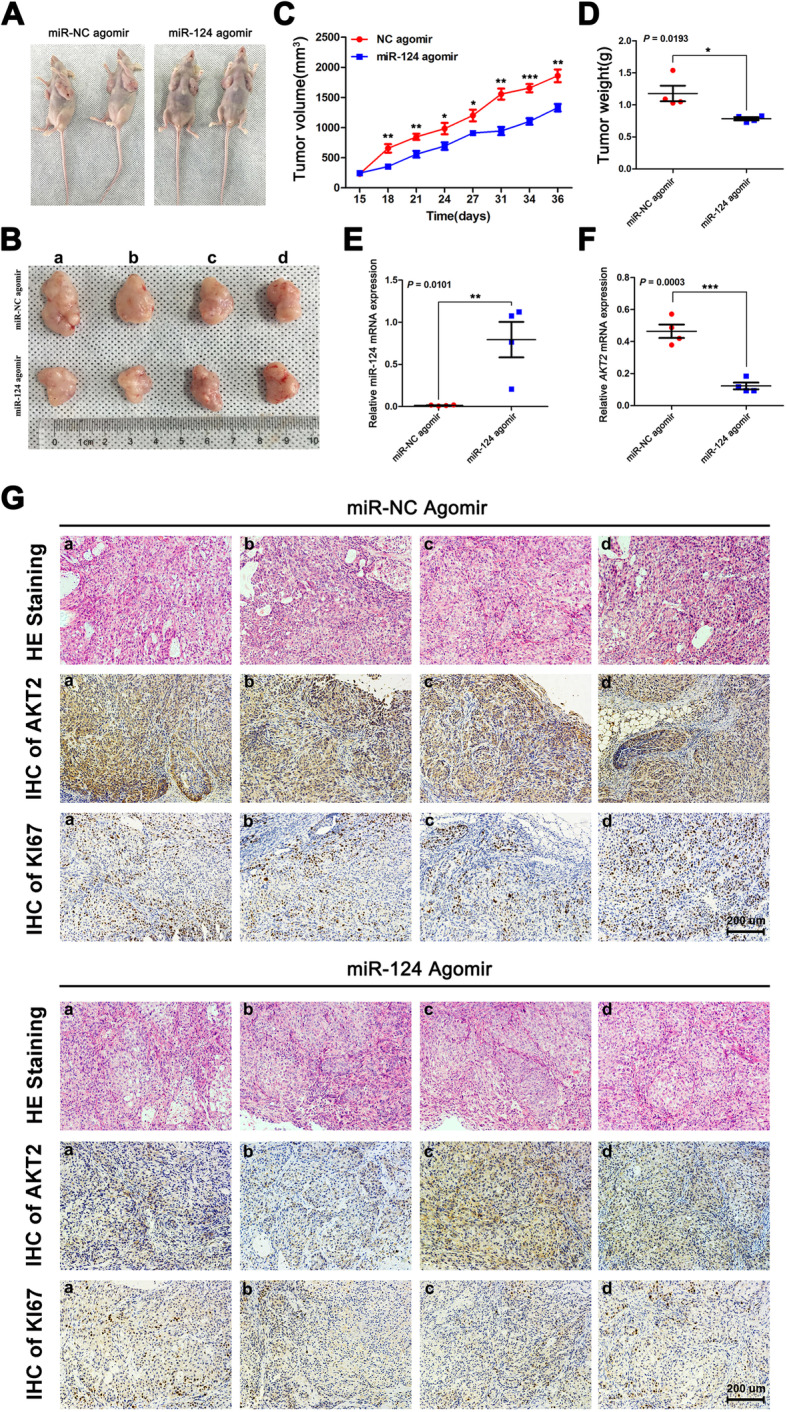
MiR-124 inhibits tumor growth by targeting AKT2 in vivo. **a** and **b** 2 × 10^6^ A549 cells were injected into nude mice (*n* = 2 mice per group). After tumor formation, miR-124 agomir and miR-NC agomir were injected into each tumor. Thirty-six days later, mice were euthanasia and tumors were surgically resected. Representative images of mice (**a**) and tumors (**b**) are shown. **c** The xenograft tumor volumes were measured every 2–3 days for total 36 days. Graph of tumor growth curves at the experimental endpoint. **d** Quantification of tumor weights from control and miR-124 agomir group. **e** and **f** qRT-PCR analysis of miR-124 and *AKT2* mRNA expression in each excised tumor. *U6* and *ACTB* were used as internal controls, respectively. **g** Representative images of H&E staining and IHC staining show tumor cells and the expression of AKT2 and KI67 in excised tumors from control or miR-124 agomir group. Scale bar, 200 um. *P < 0.05 **P < 0.01 ***P < 0.001

Finally, to further investigate the effect of miR-124 on the proliferation of LUAD cells in vivo, A549 cells were subcutaneously injected into athymic BALB/C mice, followed by intratumoral treatment with a miR-124 agomir or miR-NC agomir (Fig. 7a). Tumor growth and increases in tumor weight were sharply inhibited in the miR-124 agomir treatment group compared with the control group (Fig. 7b-d). At 36 days after tumor cell infection, we sacrificed the mice and excised the xenograft tumors. QRT-PCR was used to evaluate the expression of miR-124 and AKT2 in these tumor tissues. The data indicated that miR-124 expression was indeed upregulated, while AKT2 expression was downregulated in the miR-124 agomir treatment group (Fig. 7e and f). Moreover, an immunohistochemical assay was used to evaluate AKT2 and KI67 expression levels in these tumors (Fig. 7g). Compared with the control group, the miR-124 agomir group showed lower protein expression of both AKT2 and KI67. The results also confirmed that miR-124 overexpression could inhibit tumor growth by suppressing the expression of AKT2 in vivo.

## Discussion

AKT2, characterized as a serine/threonine protein kinase, has been proven to facilitate and contribute to tumor development in addition to its roles in angiogenesis, myoblast differentiation, glucose metabolism and inflammatory disease regulation [24]. AKT2 has been discovered to be overexpressed in human cancer tissues, including lung cancer, breast cancer, and colon cancer tissues [25–27]. An increasing amount of evidence supports that AKT2 can be regarded as a prognostic indicator in cancer patients. In osteosarcoma, AKT2 expression is significantly higher in cancerous tissues than in noncancerous tissues, implying shortened event-free survival and overall survival [28]. In meningiomas, AKT2 expression is negatively correlated with patient recurrence-free survival [29]. In pancreatic cancer, elevated AKT2 expression indicates shortened progression-free survival and overall survival [30]. In our present study, we confirmed that AKT2 is highly expressed in LUAD and closely related to the survival of LUAD patients. Although AKT2 is also highly expressed in lung squamous cell carcinoma, our study found that it was not related to the prognosis of lung squamous cell carcinoma, partially owing to the small sample size of evaluated patients. On the other hand, there might be other factors that contributed to the differences between the LUAD and LUSC subgroups, but this point needs further study.

As an important downstream component of the PI3K pathway, activated AKT2 can promote the transcription of the downstream substrate rapamycin (mTOR) and transcription factors Forkhead family (FOXO), which is involved in protein synthesis, cell proliferation, metastasis and survival processes [31]. Our study showed that A549 and H1299 cells contained the highest AKT2 expression levels among four adenocarcinoma cell lines tested. Therefore, we chose A549 and H1299 cells to explore the role of AKT2 in LUAD. We silenced AKT2 expression with specific siRNAs in these two cell lines. The results showed that silencing AKT2 expression decreased cell proliferation via cell cycle arrest, while apoptosis was not affected. Moreover, migratory and invasive abilities were inhibited in the AKT2-knockdown group.

Although the role of AKT2 in human cancer has been well established, the specific mechanism of AKT2-mediated tumor development remains to be explored. Studies have demonstrated that activated AKT2 can result in transcriptional control, such as the suppression of p21, Bax, Bad and procaspase-9 expression or the promotion of insulin-like growth factor receptor-1 expression [9, 32–35]. Our data showed that the mRNA and protein expression levels of EMT markers (N-cadherin, Vimentin and Slug), MMP7, p-AKT, and p-Erk were consistently decreased after AKT2 knockdown in LUAD cells. Therefore, the results of our current research illustrate the role of AKT2 as a tumor oncogene, promoting cell proliferation and metastasis in LUAD.

Previous studies have proven that AKT2 can be mainly regulated by upstream PI3K and PTEN. In contrast to the stimulatory role of PI3K, PTEN negatively regulates the AKT2 signaling pathway [35]. Apart from this signaling pathway, AKT2 can also be regulated by microRNAs [36]. MicroRNA/mRNA interactions are regarded as a fundamental and epigenetic gene-regulatory mechanism. Based on the analysis of four bioinformatic databases including miRDB, miRWalk, miRTarBase and TargetScan, seven microRNAs were predicted to potentially bind to AKT2. MiR-124 was found to potentially contribute to AKT2 overexpression. MiR-124 has been reported to play critical roles in tumor formation and development in multiple cancers, such as lung, breast and liver cancer [16, 37, 38]. In liver cancer, miR-124 overexpression reduces the expression of CLIC1, thereby reducing the migration and invasion of liver cancer cells [38]. In lung adenocarcinoma, miR-124 regulates cell proliferation, migration, and invasion by directly targeting SOX9 [23]. However, the potential association between miR-124 and AKT2 in LUAD is unclear. In the current study, we first proved that miR-124 can bind to the wild-type 3′-UTR but not a mutant 3′-UTR via a luciferase assay. Additionally, we found that the expression of miR-124 in NSCLC tissues was lower than that in normal lung tissues and negatively correlated with AKT2 expression. Then, we overexpressed miR-124 in A549 and H1299 cells to evaluate its effects on proliferation, metastasis and invasion. Our results showed that AKT2 expression could be significantly decreased by miR-124 overexpression. Consistently, CCK-8 and transwell assays confirmed that miR-124 overexpression could significantly reduce cell proliferation, metastasis and invasion. Furthermore, we found that upregulated miR-124 expression could inhibit the expression of EMT markers, MMPs, p-AKT, and p-Erk. Finally, our experimental results were verified in vivo*.*

## Conclusions

In summary, this study found that AKT2 was highly expressed in LUAD and promoted cell growth, metastasis and invasion. MiR-124 overexpression could negatively regulate AKT2 by binding to the 3′-UTR of AKT2 mRNA and thus inhibit the occurrence and development of LUAD. Therefore, the miR-124/AKT2 axis may serve as a potential target for novel therapies for LUAD.

## Supplementary information

**Additional file 1: Table S1.** The sequences of primers used in our study.

**Additional file 2: Table S2.** Various clinical characteristics and the mRNA expression levels of AKT2 and miR-124 in NSCLC tissues.

**Additional file 3.** Supplementary original blots.

## Data Availability

The datasets used and/or analyzed during the current study are available from the corresponding author on reasonable request.

## Abbreviations

NSCLC: Non-small cell lung cancer
LUAD: Lung adenocarcinoma
LUSC: Lung squamous cell carcinoma
qRT-PCR: Quantitative real-time PCR
HE staining: Hematoxylin and eosin (HE) staining
IHC: Immunohistochemistry
CCK-8: Cell counting kit-8
EMT: Epithelial-mesenchymal transition
MMPs: Matrix metalloproteinases
3′-UTR: 3′-Untranslated region
TIMER database: Tumor Immune Estimation Resource database
OS: Overall survival
PFS: Progression-free survival
PTEN: Phosphatase and tensin homologue
NC: Negative control
NS: No significance
