# Supplementary material for: AKT2 drives cancer progression and is negatively modulated by miR-124 in human lung adenocarcinoma
Source: Respir Res. 2020 Sep 1;21:227. doi: 10.1186/s12931-020-01491-0 (PMC7466426; doi:10.1186/s12931-020-01491-0)
Supplement: Supplementary file 1 — Additional file 1: Table S1. The sequences of primers used in our study. [file 12931_2020_1491_MOESM1_ESM.docx]

| **Name** | **position** | **Sequences (**5'‑3'**)** |
| --- | --- | --- |
| *AKT2* | FP | GCAAAGAGGGCATCAGTGAC |
|  | RP | CTCGTGGTCCTGGTTGTAGA |
| *CDH1* | FP | CGAAAGGCCTTCAACTGCAAAT |
|  | RP | ACTGGTACTTCTTGACATCTG |
| *CDH2* | FP | TGGGAATCCGACGAATGG |
|  | RP | TGCAGATCGGACCGGATACT |
| *VIM* | FP | TGAGTACCGGAGACAGGTGCAG |
|  | RP | TAGCAGCTTCAACGGCAAAGTTC |
| *SNAI1* | FP | CGAAAGGCCTTCAACTGCAAAT |
|  | RP | ACTGGTACTTCTTGACATCTG |
| *SNAI2* | FP | TGTTGCAGTGAGGGCAAGAA |
|  | RP | GACCCTGGTTGCTTCAAGGA |
| *MMP7* | FP | ATGTTAAACTCCCGCGTCATA |
|  | RP | CAGCATACAGGAAGTTAATCC |
| *MMP9* | FP | AGACCTGGGCAGATTCCAAAC |
|  | RP | CGGCAAGTCTTCCGAGTAGT |
| *ZEB1* | FP | TTCAAACCCATAGTGGTTGCT |
|  | RP | TGGGAGCACCAAACCAACTG |
| *ZEB2* | FP | ACTTTTCCTGCCCTCTCTGT |
|  | RP | TTGCGATTACCTGCTCCTT |
| *ACTB* | FP | CACAGAGCCTCGCCTTTGCC' |
|  | RP | ACCCATGCCCACCATCACG |

**Table S1.** The sequences of primers involved in our study
