# Supplementary material for: AKT2 drives cancer progression and is negatively modulated by miR-124 in human lung adenocarcinoma
Source: Respir Res. 2020 Sep 1;21:227. doi: 10.1186/s12931-020-01491-0 (PMC7466426; doi:10.1186/s12931-020-01491-0)
Supplement: Supplementary file 2 — Additional file 2: Table S2. Various clinical characteristics and the mRNA expression levels of AKT2 and miR-124 in NSCLC tissues. [file 12931_2020_1491_MOESM2_ESM.docx]

**Table S2.** Various clinical characteristics and mRNA expression levels of *AKT2* and miR-124 in NSCLC tissues

| Characteristics | n (%) | *AKT2* mRNA expression | miR-124 mRNA expression | |
| --- | --- | --- | --- | --- |
| Age |  |  | |  |
| ≥65 | 22(48.8%) | 0.09927 ± 0.02420 | | 0.0003891 ± 0.0001980 |
| ＜65  *P* value | 23(51.2%) | 0.2086 ± 0.06895  0.1462 | | 0.0003893 ± 0.0001513 0.9995 |
| Gender |  |  | |  |
| Male | 27(60.0%) | 0.2077 ± 0.06013 | | 0.000472 ± 0.000195 |
| Female  *P* value | 18(40.0%) | 0.07627 ± 0.01673  0.0439 | | 0.000264 ± 0.00009  0.3400 |
| Histology |  |  |  | |
| Adenocarcinomas | 27(60.0%) | 0.1649 ± 0.05974 | 0.0004194 ± 0.0001380 | |
| Squamous cell carcinomas  Others  *P* value | 9(20.0%)  9(20.0%) | 0.1669 ± 0.05379  0.1141 ± 0.03304  0.4211 | 0.0006524 ± 0.0004498 0.0000353 ± 0.00000873  0.3898 | |
| Lymph node |  |  |  | |
| Yes | 20(44.4%) | 0.1547 ± 0.04210 | 0.0002218 ± 0.00009327 | |
| No  *P* value | 25(55.6%) | 0.1555 ± 0.05968 0.9918 | 0.0005231 ± 0.0002057 | |
|  |  |  | 0.1915 | |
| Distant metastases |  |  |  | |
| Yes | 3(6.7%) | 0.1942 ± 0.09554 | 0.0005880 ± 0.0005570 | |
| No  *P* value | 42(93.3%) | 0.1524 ± 0.03997  0.7251 | 0.0003750 ± 0.0001269 | |
|  |  |  | 0.7450 | |
| TNM stage |  |  |  | |
| Ⅰ | 19(42.2%) | 0.1716 ± 0.07680 | 0.0005886 ± 0.0002579 | |
| Ⅱ | 5(11.1%) | 0.04971 ± 0.0097 | 0.0000359 ± 0.0000162 | |
| Ⅲ | 18(40.0%) | 0.1606 ± 0.04683 | 0.0002437 ± 0.0001026 | |
| Ⅳ  *P* value | 3(6.7%) | 0.1942 ± 0.09554 | 0.0005880 ± 0.0005570 | |
|  |  | 0.4542 | 0.2664 | |

Date are presented as mean ± SEM. Unpaired t test for 2 groups. Kruskal-wallis test for 3 or more groups
