# Supplementary material for: AKT2 drives cancer progression and is negatively modulated by miR-124 in human lung adenocarcinoma
Source: Respir Res. 2020 Sep 1;21:227. doi: 10.1186/s12931-020-01491-0 (PMC7466426; doi:10.1186/s12931-020-01491-0)
Supplement: Supplementary file 3 — Additional file 3. Supplementary original blots. [file 12931_2020_1491_MOESM3_ESM.docx]

| **Name** | **Molecular Weight(kDa)** |
| --- | --- |
| AKT2 | 56 |
| N-cadherin | 140 |
| Vimentin | 57 |
| Slug | 30 |
| MMP2 | 64, 72 |
| MMP7 | 22, 24 |
| MMP9 | 84, 92 |
| p-AKT | 60 |
| AKT | 60 |
| p-Erk | 42, 44 |
| Erk | 42, 44 |
| β-actin | 43 |

The molecular weights of all the proteins involved in this study

**All original uncropped and unadjusted blots**

**Figure 1B(8 NSCLC tissues with higher expression of AKT2 typically were chosen to be presented)**

**
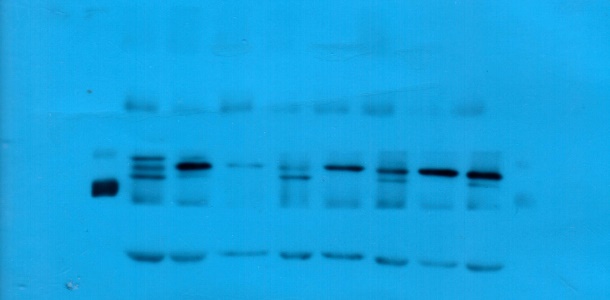

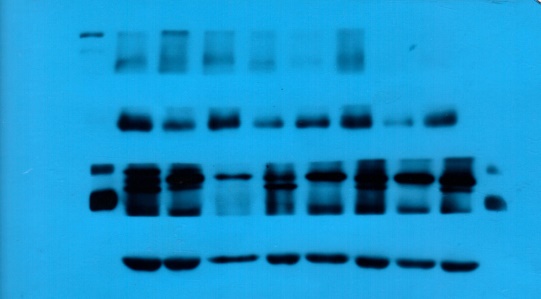
**

**AKT2**

**1N 1T**

**β-actin**

**long exposure**

**
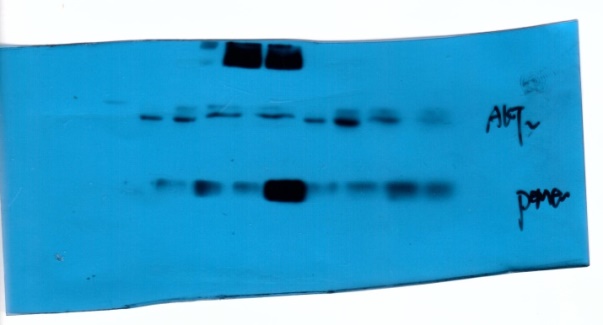

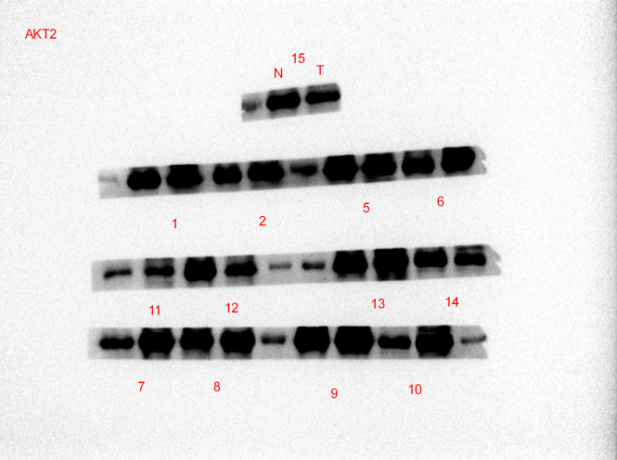
**

**AKT2**

**2N 2T**

**8N 8T**

**AKT2**

**
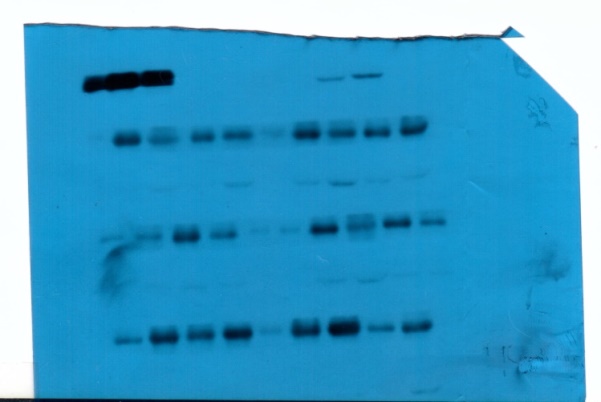

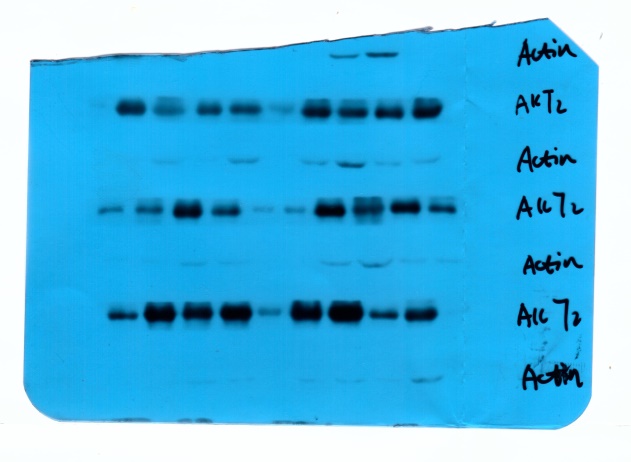
**

**AKT2**

**AKT2**

**7N 7T**

**6N 6T**

**5N 5T**

**4N 4T**

**3N 3T**

**long exposure**

**
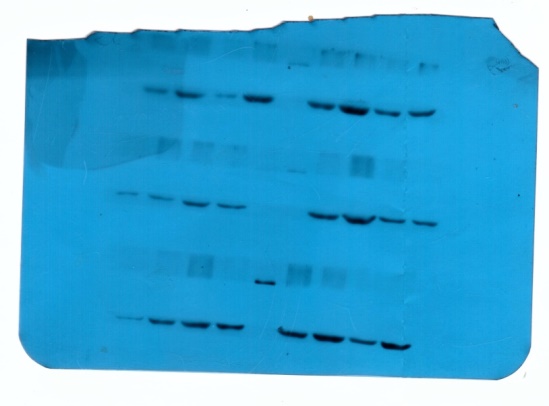

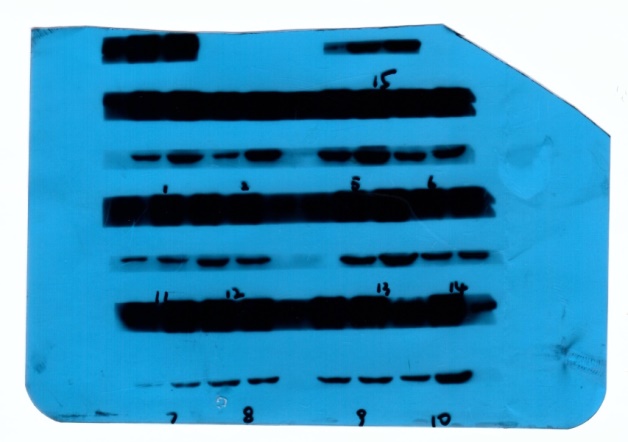
**

V

­

­

V

­

**long exposure**

**β-actin**

**β-actin**

**
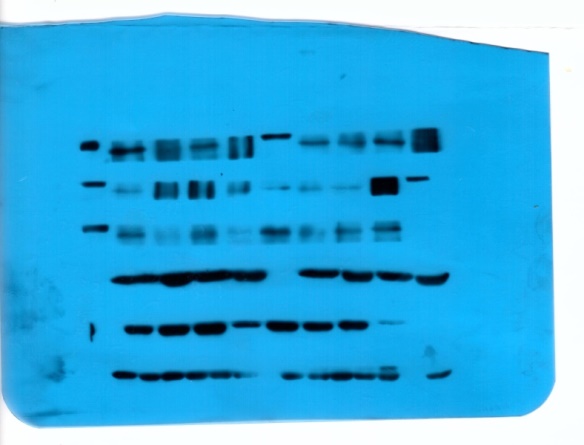
**

**β-actin**

**Figure 3A : BEAS-2B A549 H1299 LTEP-α-2 SPC-A1 H226** **SK-MES-1 H520**

Actually we have examined AKT2 expression in both lung adenocarcinoma cell lines (A549, H1299,LTEP- α-2,SPC-A1) and lung squamous cell lines (H226, SK-MES-1,H520), and we found that AKT2 protein level was higher in NSCLC cell lines when compared to BEAS-2B. However based on the Kaplan-Meier survival curves, we found that higher AKT2 expression indicated poorer overall survival in lung adenocarcinoma patients not in lung squamous cell carcinoma. So this study mainly discussed the role of AKT2 in the development of lung adenocarcinoma. Therefore, only 4 lung adenocarcinoma lines were shown in the figure 3A.


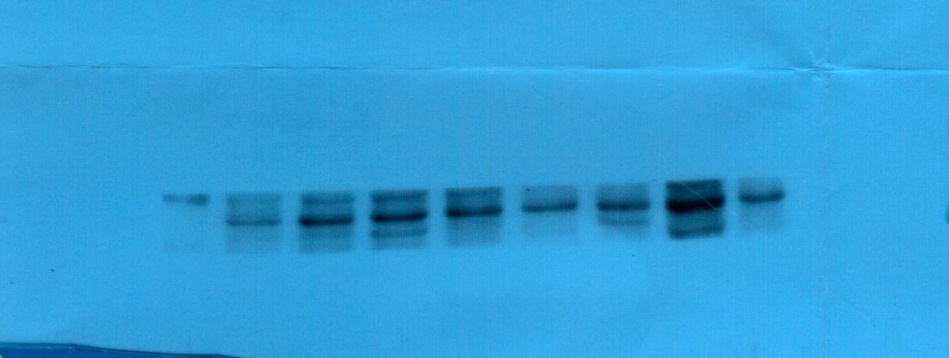

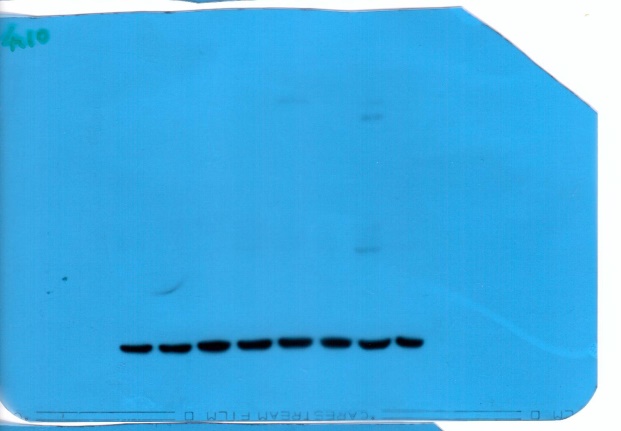


**SPC-A1**

**LTEP-α-2**

**H1299**

**A549**

**BEAS-2B**

**β-actin**

**AKT2**

**Figure 3C: si-NC si-AKT2-1 si-AKT2-2 si-AKT2-3**

**
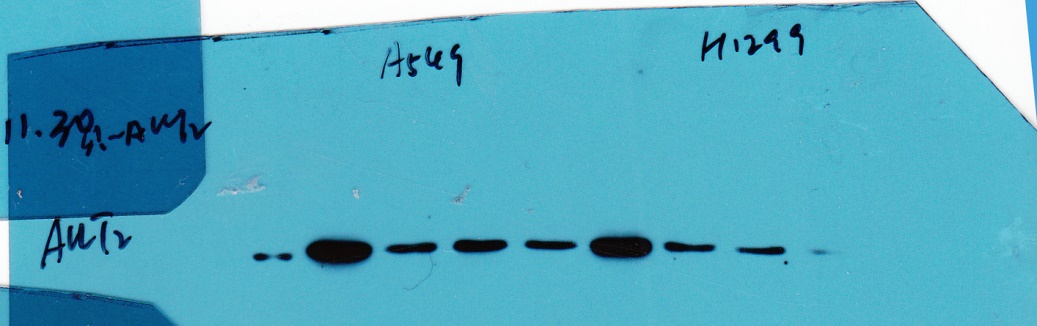
**

**AKT2**

**
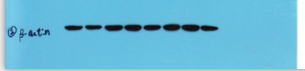

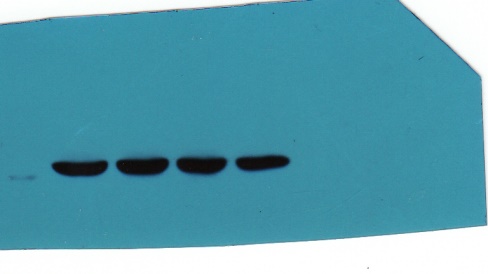
**

**A549**

**H1299**

**β-actin**

**Figure 5C, 6M**

**A549:** **si-NC si-AKT2-1 si-AKT2-3 miR-NC miR-124**

**miR-124**

**miR-NC**

**Si-ATK2-3**

**Si-ATK2-1**

**
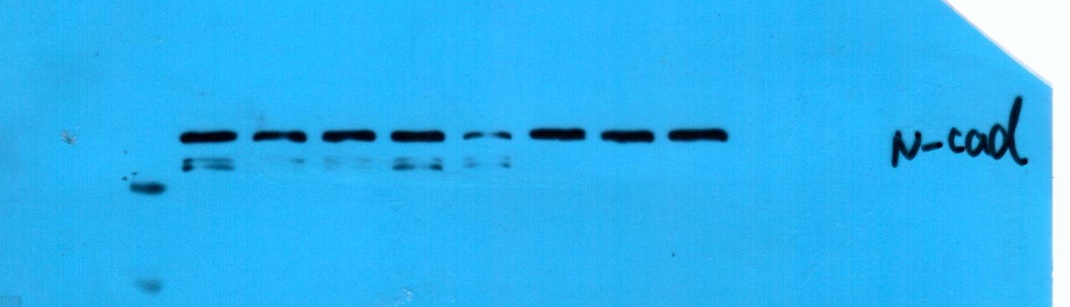
**

**Si-NC**

**N-cadherin**

**
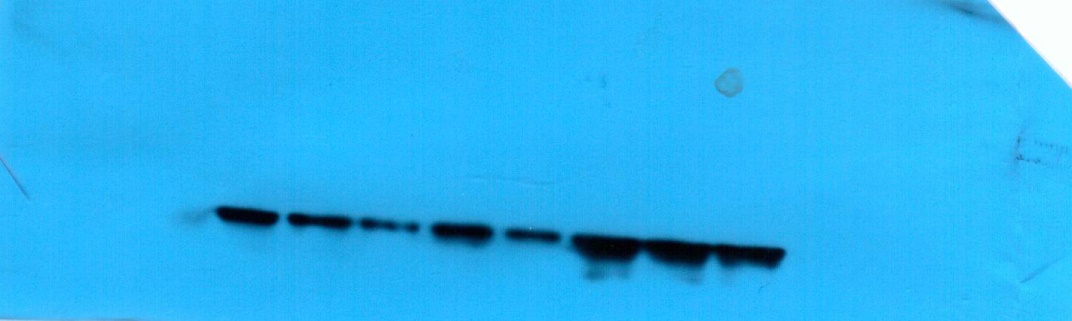
**

**Vimentin**


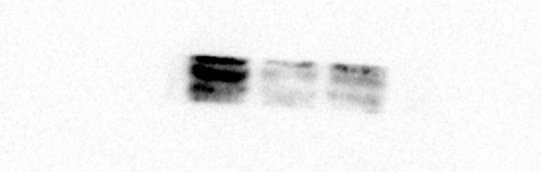
**
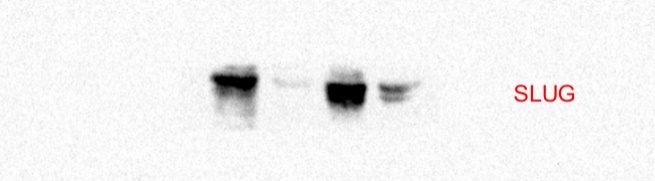
**

**Slug**

**A549:** **si-NC si-AKT2-1 si-AKT2-3 miR-NC miR-124**

**
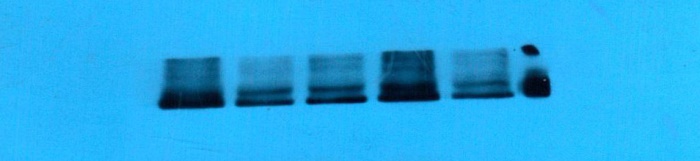
**

**MMP2**


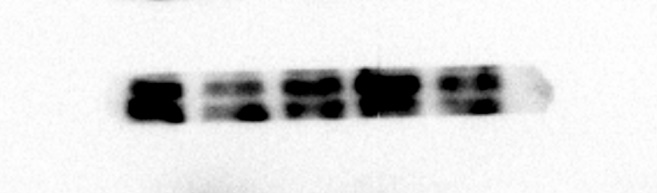
**
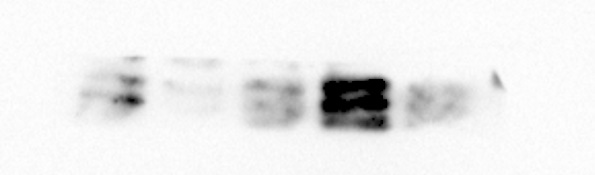
**

**MMP7**

**
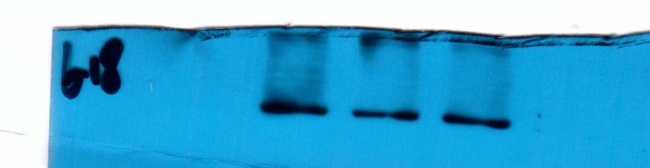

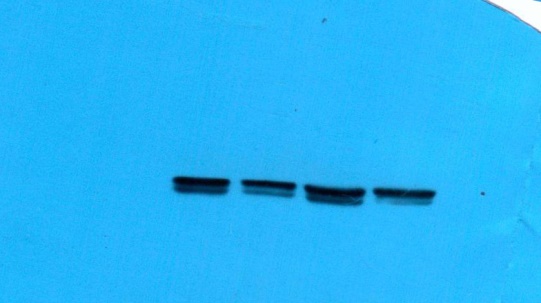
**

**MMP9**

**
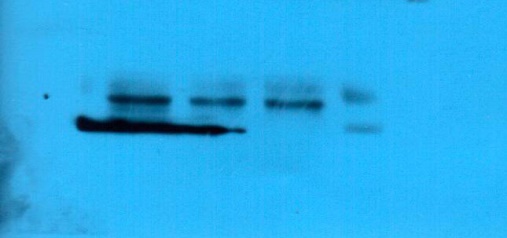

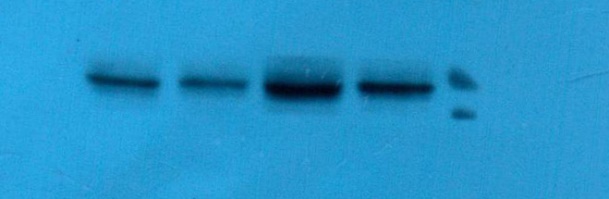
**

**p-AKT**

**
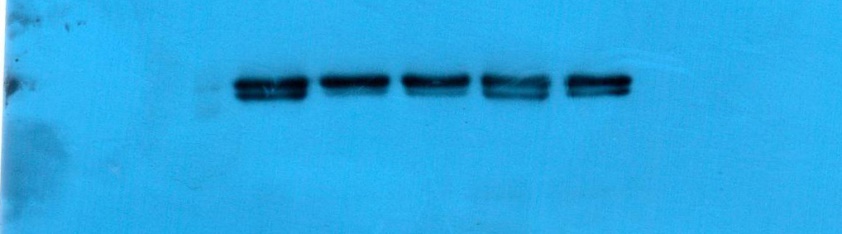
**

**AKT**

**
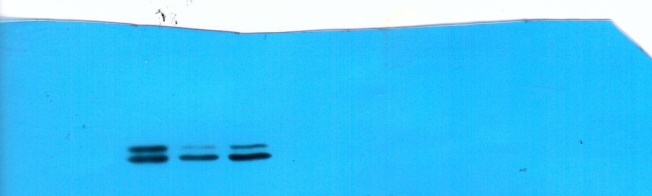

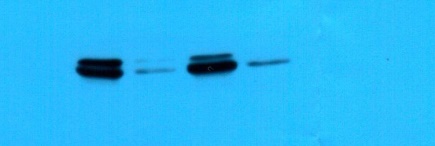
**

**p-Erk**


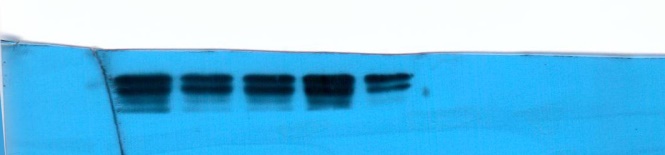
**
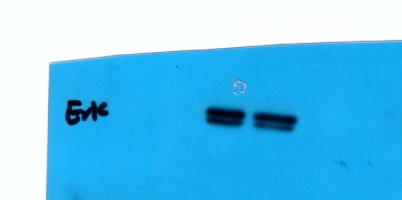
**

**Erk**

**
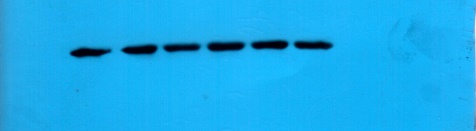

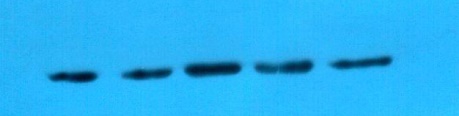
**

**β-actin**

**H1299: si-NC si-AKT2-1 si-AKT2-3 miR-NC miR-124**

**miR-NC**

**Si-ATK2-3**

**Si-ATK2-1**

**Si-NC**

**miR-124**

**
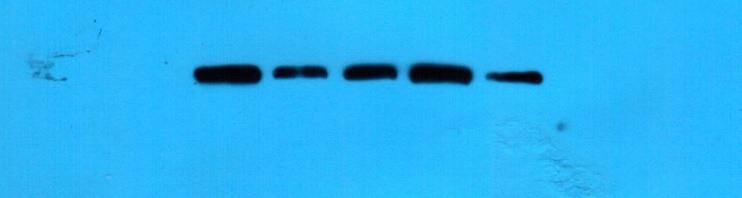
**

**N-cadherin**

**
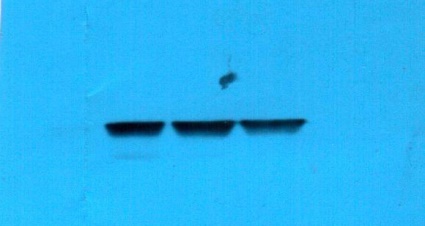

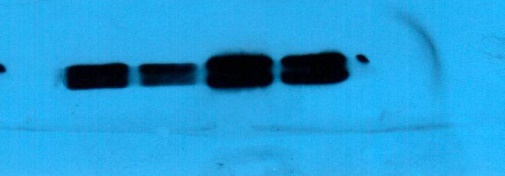
**

**Vimentin**

**
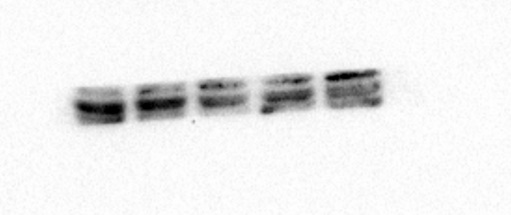

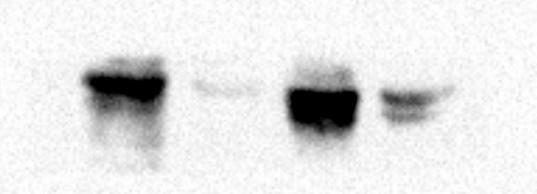
**

**Slug**


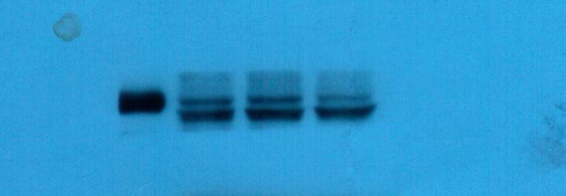

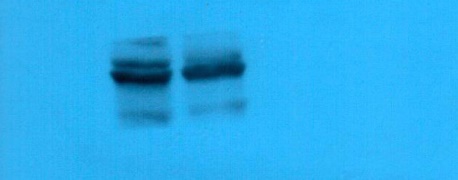


**MMP2**

**
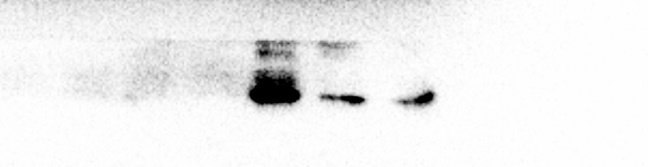

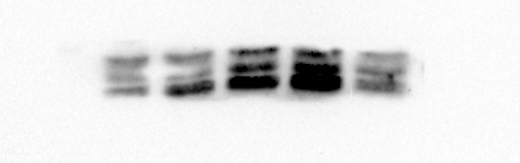
**

**MMP7**

**
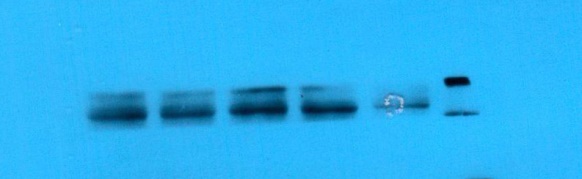
**

**MMP9**

**
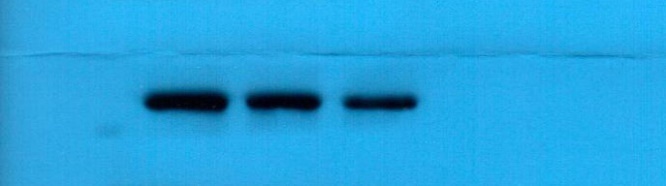

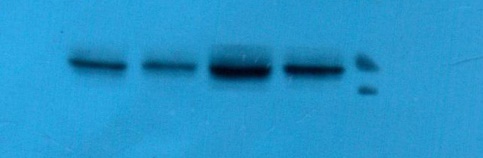
**

**p-AKT**

**H1299: si-NC si-AKT2-1 si-AKT2-3 miR-NC miR-124**

**
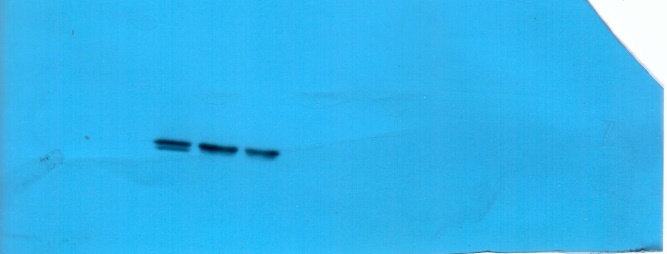

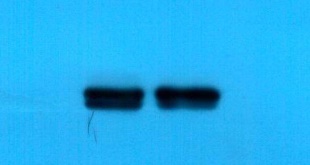
**

**AKT**

**
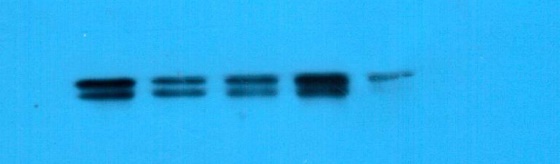

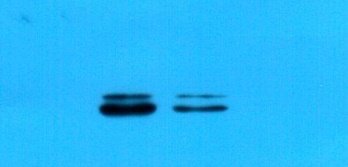
**

**p-Erk**

**
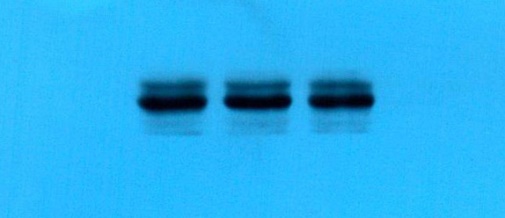

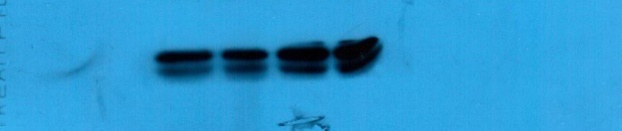
**

**Erk**

**
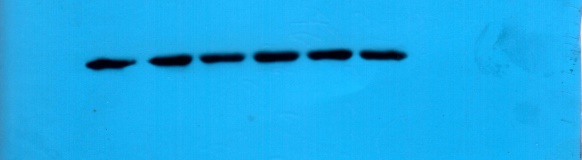

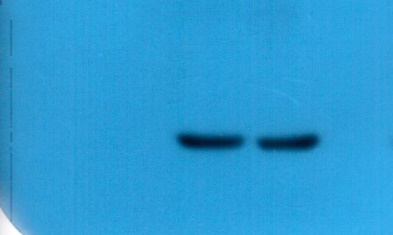
**

**β-actin**

**Erk 42 44 kDa**
